# How Deep Is Your Mud? Pinpointing Hidden Transitions in Restored Saltmarsh Sediments to Support Robust Assessment of Carbon Stocks

**DOI:** 10.1007/s12237-026-01812-4

**Published:** 2026-09-24

**Authors:** Robert B. Sparkes, Saule Akhmetkaliyeva, Stuart A. Rae, Callum Johnstone, Josie Tustain, Amber Ferguson, Ewan Hoburn, Lucy McMahon, Rachel M. Dunk, Hannah L. Mossman

**Affiliations:** 1 Department of Natural Sciences, Manchester Metropolitan University, Chester Street, Manchester, M1 5GD UK; 2 School of Biology and Environmental Science, University College Dublin, Belfield 4, Dublin, Ireland; 3 Department of Engineering, Lancaster University, Lancaster, UK; 4 Wetland Evidence, WWT, Slimbridge, Gloucestershire UK

**Keywords:** Blue carbon, Saltmarsh, Monitoring reporting and verification, Biomarkers, Soil horizon, Managed realignment

## Abstract

Tidal inundation of saltmarshes can mitigate anthropogenic carbon dioxide emissions by rapidly accumulating carbon-rich sediment, with the potential to sequester this “blue carbon” for thousands of years. The desire to remove atmospheric carbon, alongside increased flood risk from sea-level rise, is driving scientific and financial interest in restoring low-lying land into saltmarshes. Saltmarsh restoration buries agricultural fields with marine or estuarine sediment, yet the transition from agricultural soil to saltmarsh sediment is often difficult to observe in sediment cores. Quantifying sediment accumulation is critical for calculating the carbon benefits of saltmarsh restoration, and a pre-requisite for generating financial returns. At Bleadon Levels, a restored saltmarsh in Somerset, UK, we tested several methods to determine the transition between agricultural soils and saltmarsh sediments. Calcium (Ca) and inorganic carbon (IC) were significantly more concentrated in the upper parts of cores, interpreted as marine carbonates within saltmarsh sediment deposits. The biomarker-based R_soil_ index agreed with Ca and IC data. Organic carbon (OC) measurements did not show matching changes, demonstrating that OC variations with depth do not reliably identify transitions from saltmarsh to agricultural layers. Thus, saltmarsh sediment depth can be determined using relatively simple, cost-effective Ca and IC concentration measurements, and restoration schemes should consider these techniques in their monitoring, reporting and verification procedures.

**Supplementary Information:**

The online version contains supplementary material available at https://doi.org/10.1007/s12237-026-01812-4.

## Introduction

Coastal ecosystems, including saltmarshes, are carbon dense and may accumulate carbon an order of magnitude faster than terrestrial ecosystems (Mcleod et al., 2011; Mossman et al., 2022). Saltmarshes capture and store carbon that originates both within (autochthonous) and outside of the habitat (allochthonous); carbon accumulates in saltmarshes as sediment carried in by the tide is deposited, and this sediment, which can be rich in allochthonous carbon, buries saltmarsh plant litter and roots (Ouyang et al., 2017; Ouyang et al., 2023; Van de Broek et al., 2018). Thus, the creation and restoration of saltmarshes is often a focal point of blue carbon strategies as an approach to mitigate against the causes of climate change (Macreadie et al., 2021; Serrano et al., 2019; Vieira da Silva et al., 2014), while also mitigating climate change effects by providing flood protection (Scott et al., 2016). Restoration of saltmarsh via managed realignment, the deliberate breaching of coastal defences onto (often low-lying) agricultural land, provides large amounts of accommodation space for coastal sediment, which rapidly accumulates (Gore et al., 2026; Mossman et al., 2012). Pre-existing agricultural soils can be buried under tens of centimetres of carbon-rich marine sediment in only a few years (Mossman et al., 2022; Wollenberg et al., 2018). Since the mass of carbon buried is related to the volume of sediment deposited, accurate measurements of sediment accumulation are required to understand the potential benefits of saltmarsh restoration.

### Measuring Sediment Depth to Support Monitoring, Reporting and Verification of Carbon Burial

Global efforts to promote and support the restoration of coastal ecosystems have led to the development of carbon accountancy procedures, which rely on the accurate and precise quantification of carbon burial (Burden et al., 2023; Emmer et al., 2023; Lovelock et al., 2023). Alongside measurements of carbon concentration, the monitoring and auditing of restored saltmarshes requires robust and cost-effective techniques for determining sediment accumulation depths. Remote sensing techniques, such as aerial LiDAR, provide good spatial coverage but need referencing against the pre-restoration surface. They also require calibration via fixed reference points, and the growth of vegetation can introduce uncertainty (Fagherazzi et al., 2020; Goodwin & Mudd, 2019; Hladik et al., 2013). In-situ sediment deposition markers (e.g., sediment pins, feldspar marker horizons, solid plates) must be installed before restoration begins and can be washed away by the tide, disrupted by bioturbation, or lost if sedimentation is very high (Nolte et al., 2013; Oosterlee et al., 2020). Additionally, none of these techniques provide an indication of the carbon stored without additional laboratory measurements, nor can they be applied without pre-restoration surveys. Without careful planning prior to restoration, post-hoc methods of determining sedimentation rate are required, using sediment cores collected for analysis of carbon content. Visual inspection of sediment cores can struggle to distinguish between pre-restoration soils and post-restoration deposition, but more advanced methods can be used to look for the transition.

Organic carbon (OC) measurements are essential when calculating the climate mitigation potential of a restored saltmarsh (Mossman et al., 2022), and are a foundational part of the monitoring, reporting and verification process for audited saltmarsh restoration. Typically, restored saltmarsh sediments have a different OC concentration to the pre-restoration soils (Gore et al., 2026; Mossman et al., 2022). If abrupt down-core changes in organic carbon content reliably showed the transition from agricultural soil to saltmarsh sediment, the depth of sediment deposited within restored saltmarshes could be verified without additional expenditure. However, degradation of OC within the saltmarsh sediments, altering down-core OC concentrations, would compromise this (Piñeiro Juncal et al., 2025).

Stable carbon and nitrogen isotopes have been used to infer organic matter sources in saltmarshes, including terrestrial/saltmarsh vegetation and marine sources (Smeaton et al., 2023). A sharp transition from terrestrial to marine isotope signatures would imply the breach horizon, but this was not consistently seen in the study by Gore et al. (2026) who found carbon isotope ratios were higher in the new sediment at one site, lower at another, and showed no change at a third. Thus, the tendency for organic matter to transform or degrade after burial could limit the applicability of organic carbon concentration or isotopes as a marker for down-core transitions.

Radiometric dating (^210^Pb and ^137^Cs) has also been used to estimate horizon depth and has the advantage of generating an age-depth model which can be compared to historical records. However, the technique is limited by saltmarsh age, since young marshes do not produce interpretable isotope profiles (Gore et al., 2026). The amount and type of foraminifera have been used to infer breach depth—assuming that foraminifera would only be present after the site was breached—with success in some sites but not others (Gore et al., 2026).

Bacteriohopanepolyol biomarkers (BHPs) are ubiquitous bacterial molecules found throughout lakes, rivers, oceans, soils and peat. They control cell membrane rigidity and permeability, performing a variety of functions including lipid ordering and antibiotic, pH, temperature, and detergent resistance (Kusch & Rush, 2022). Comparison of the bacteriohopanetetrol molecule (BHT), typically used as a marker for marine organic matter, with nucleoside BHPs (also known as soil marker BHPs) allows estimation of the relative input of terrestrial vs. aquatic organic matter in coastal and marine sediments via the R_soil_ index which has usually demonstrated good, often linear, agreement with other terrestrial-marine proxies (Akhmetkaliyeva et al., 2025; Bischoff et al., 2016; Cooke et al., 2008b; Doğrul Selver et al., 2012; Kusch & Rush, 2022; Talbot & Farrimond, 2007; Zhu et al., 2011).

Major elements, such as calcium, aluminium and silicon, are primary components of carbonates, clay minerals and sand respectively (Zwolsman et al., 1993). Some trace metals (e.g. Cd, Pb) are soluble and mobile in saline sediments, but others (e.g. Zn) remain adsorbed to minerals (Acosta et al., 2011), and redox conditions within saltmarshes determine their dissolution and precipitation (Zwolsman et al., 1993). However, transport, storage and preparation protocols for elemental geochemistry are typically simple, and measurements are commercially available at relatively low cost. If elemental concentrations vary systematically between saltmarsh and agricultural sediments, their analysis may provide a cost-effective method for measuring sediment accumulation and identifying sediment transitions.

### Aims and Objectives

There is a scientific, regulatory and financial need to robustly qualify the amount of sediment deposited in restored saltmarshes. Therefore, this study aimed to test and compare a variety of methods for identifying the depth of the transition between pre-restoration agricultural soils and post-restoration saltmarsh sediment.

We collected and analysed sediment cores from Bleadon Levels, a restored saltmarsh in Somerset, UK. Sediments from locations within the restored saltmarsh site were analysed using organic and inorganic carbon fractions, major and trace element concentrations, and BHP biomarkers. Down-core trends and relationships were explored to determine the most reliable proxies and indicators for defining a sample as agricultural or saltmarsh in origin and to make recommendations for measuring post-restoration sediment accumulation.

## Methods

### Sample Site

**Fig. 1:**
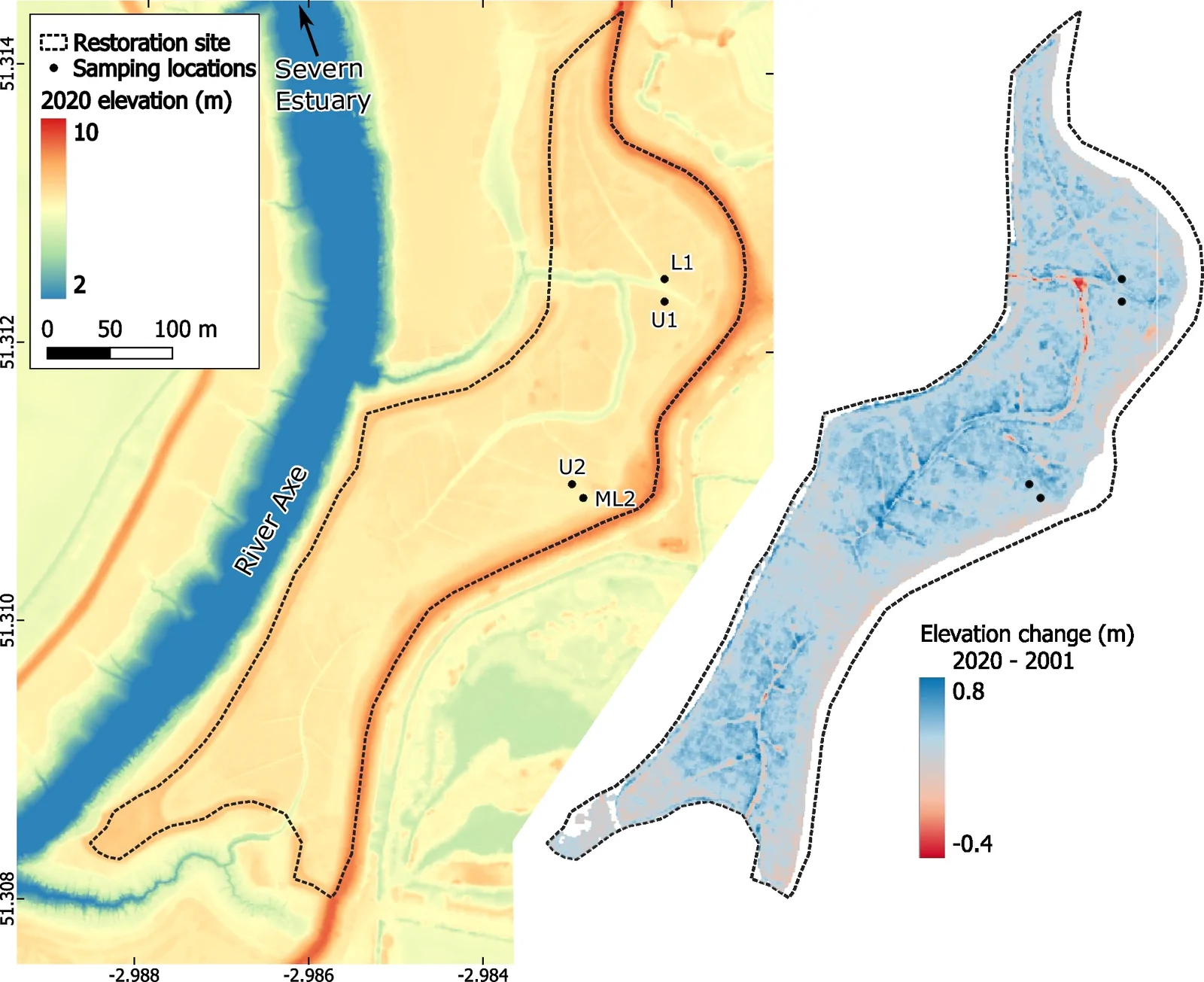
Topographic and elevation change maps of the Bleadon Levels restored saltmarsh site. Topographic map (left side) shows a LiDAR-derived digital terrain model (DTM) from a survey in 2020. Elevation change map (right side) shows the difference between DTMs derived from LiDAR surveys from 2020 and 2001. Core locations are shown as black dots and are labelled in the topographic map. Restoration site boundary is shown as a dashed line

Bleadon Levels is located on the River Axe, a tributary of the Severn Estuary, south-west England (51.3110, −2.9846). The bedrock of the area is part of the Mercia Mudstone Group, consisting of Triassic mudstones and halites, overlain by tidal flat deposits (British Geological Survey, 2025). The restoration site comprised 13 hectares of livestock grazed grassland, restored to saltmarsh in 2001 via the digging of a creek network followed by multiple breaches of the pre-existing sea defences, allowing tidal inundation (Fig. 1). Due to the relatively high position in the tidal frame, saltmarsh vegetation developed quickly (Mossman et al., 2012). Methods to reliably measure sedimentation or elevation change were not installed during restoration, so no definitive measurement of sediment accretion or erosion existed prior to this study. Estimates from aerial LiDAR, flown before restoration and in 2020, indicated that there was erosion in the creeks, but most of the site experienced deposition of up to 80 cm (Fig. 1).

### Core Sampling

Two pairs of sediment cores were collected at Bleadon Levels in January 2022 from different tidal elevations (Fig. 1): cores U1 and U2 were located on topographically high points, with vegetation dominated by *Elytrigia atherica*, typical of upper saltmarsh. Cores L1 and ML2 were located near to cores U1 and U2, respectively, but in topographically lower positions nearer to the artificial creeks, with vegetation dominated by *Puccinellia maritima* and *Tripolium pannonicum*, species common of low and mid-low saltmarsh. Cores of 40 to 50 cm were collected using a gouge soil auger (Royal Eijkelkamp, inserted until core refusal), photographed, then cut into subsamples, at ~ 5 cm intervals. No definitive colour changes or past vegetation horizons were observed during coring or in photographs, apart from slightly darker sediment in the top few cm of cores U1 and U2, suggesting development of an organic-rich layer. Samples were kept dark and refrigerated upon collection, returned to Manchester Metropolitan University within three days, and then stored in a refrigerator below 5 °C. For long term stability, surface and core samples were oven dried at 60 °C, ground with a ceramic mortar and pestle and archived in glass vials.

### Elemental Concentrations

Dried and ground samples were weighed (0.5 g) into 50 ml plastic centrifuge tubes, and 5 ml of concentrated nitric acid (68%; Primar Plus-Trace Metal Analysis grade, Fisher Scientific) added to each tube. Samples were sonicated for 10 min in an ultrasonic bath followed by one hour of further digestion. Digested supernatants were diluted to ~ 20 ml to reduce acid strength, gravity filtered (using Whatman 540 filter papers) and diluted again to 50 ml using a volumetric flask. The filtered, diluted solution was analysed using inductively coupled plasma optical emission spectroscopy (ICP-OES; iCAP6300Duo with Cetac ASX520 auto sampler, Thermo Scientific). Method parameters were as follows: UV exposure time 15 s, visible exposure time 5 s, RF power 1200 W, nebuliser gas flow 0.55 L min^−1^. Seventeen elements were quantified against a reference standard. Three replicate measurements were made for each metal in every sample, with low relative standard deviations (0.26% to 2.63%). Elemental concentrations in the solution, reported in mg L^−1^, were converted to mg per kg of dry sediment.

### Total, Inorganic and Organic Carbon Concentrations

Measuring organic carbon within sediments requires separating the total carbon (TC) into the organic (OC) and inorganic (IC) components. For this study, elemental analysis was combined with a combustion pre-treatment following the method in Sparkes et al. (2026). Two aliquots were taken from each sample: total carbon (TC) and inorganic carbon (IC). Aliquot IC was combusted at 500 °C for 12 h in a muffle furnace (Carbolite Gero AF-1100) to remove organic matter. Both aliquots were weighed into tin capsules (Elemental Microanalysis Ltd.) and the carbon content measured using an elemental analyser (vario EL cube, Elementar Ltd.) The instrument was calibrated using EDTA and Soil Standard (Clay) certified reference materials (Elemental Microanalysis Ltd.) The mass of aliquot IC before and after combustion was used to correct the initial measurement into a value for sample IC. OC was calculated by subtracting IC from TC – this method has been tested thoroughly and shown to give accurate and precise OC values (Sañé et al., 2020; Serrano et al., 2023; Sparkes et al., 2026). Whilst only one replicate was measured for each core sample in this study, method validation using multiple replicates of a similar sample gave uncertainties of ± 0.03 wt% for IC, and ± 0.1 wt% for OC (Sparkes et al., 2026).

### Lipid Biomarkers

Due to the significantly greater analytical effort and cost involved, BHP analysis was limited to a subset of the sediment samples. Lipids were extracted ultrasonically from dried and ground sediments using a modified Bligh and Dyer procedure (Akhmetkaliyeva et al., 2025; O’Beirne et al., 2022). BHPs were separated from the total lipid extract using NH_2_ solid phase extraction (SPE) chromatography (Talbot et al., 2016). Immediately prior to analysis, 5β-Pregnane-3α,20α-diol internal standard (Tokyo Chemical Industry UK Ltd.) was added to the BHP fraction, which was acetylated and analysed within one week.

BHPs were measured in triplicate via reverse phase LC–MS using an Agilent 1260 HPLC and an Agilent 6540 Q-TOF mass spectrometer equipped with an APCI source operated in positive ion mode. BHPs were identified using a combination of MS mode spectra (base peak and fragment ions) and both absolute and relative retention times (Cooke et al., 2008a; O’Beirne et al., 2022). Seventeen BHP molecular structures were identified and semi-quantitative estimates of BHP concentrations were calculated using typical response factors (Akhmetkaliyeva et al., 2025; Cooke et al., 2008a; van Winden et al., 2012).

The R_soil_ index, an estimate of soil vs. marine organic carbon in a sediment, was calculated from the sum of six nucleoside BHPs compared to BHT (Zhu et al., 2011):

$${R}_{soil}=\frac{\sum nucleoside BHPs}{\sum nucleoside BHPs+BHT}$$

### Statistical Analysis

Throughout this study, where average values are reported they are presented as mean ± one standard deviation. Correlation analysis was performed using linear regression functions in Microsoft Excel. Unpaired t-tests were used to analyse significance, also using Microsoft Excel. The differences between the saltmarsh and agricultural groups were tested using principal component analysis (PCA) of the concentration data, using the “prcomp” and “ggbiplot” functions of the R software package (v4.4.2; R Core Team, 2024; Vu & Friendly, 2024).

## Results

### Elemental Data from ICP-OES

The most abundant element from the suite of targets was calcium, with a maximum concentration of 67 000 mg kg^−1^ (Table S1). Elements with consistently low concentrations were excluded from further analysis (< 30 mg kg^−1^; As, Cd, Co, Cr, Cu, Ni). Of the remaining elements, some were typically present in higher concentrations in the lower parts of cores (Al, Fe), some were present in higher concentrations in the upper parts of cores U1, U2 and ML2, and throughout core 55 (Ca, K, Mg, Pb, Zn) and some showed no clear down-core pattern (Mn, Na, P, S).

### Carbon Data from EA

**Fig. 2:**
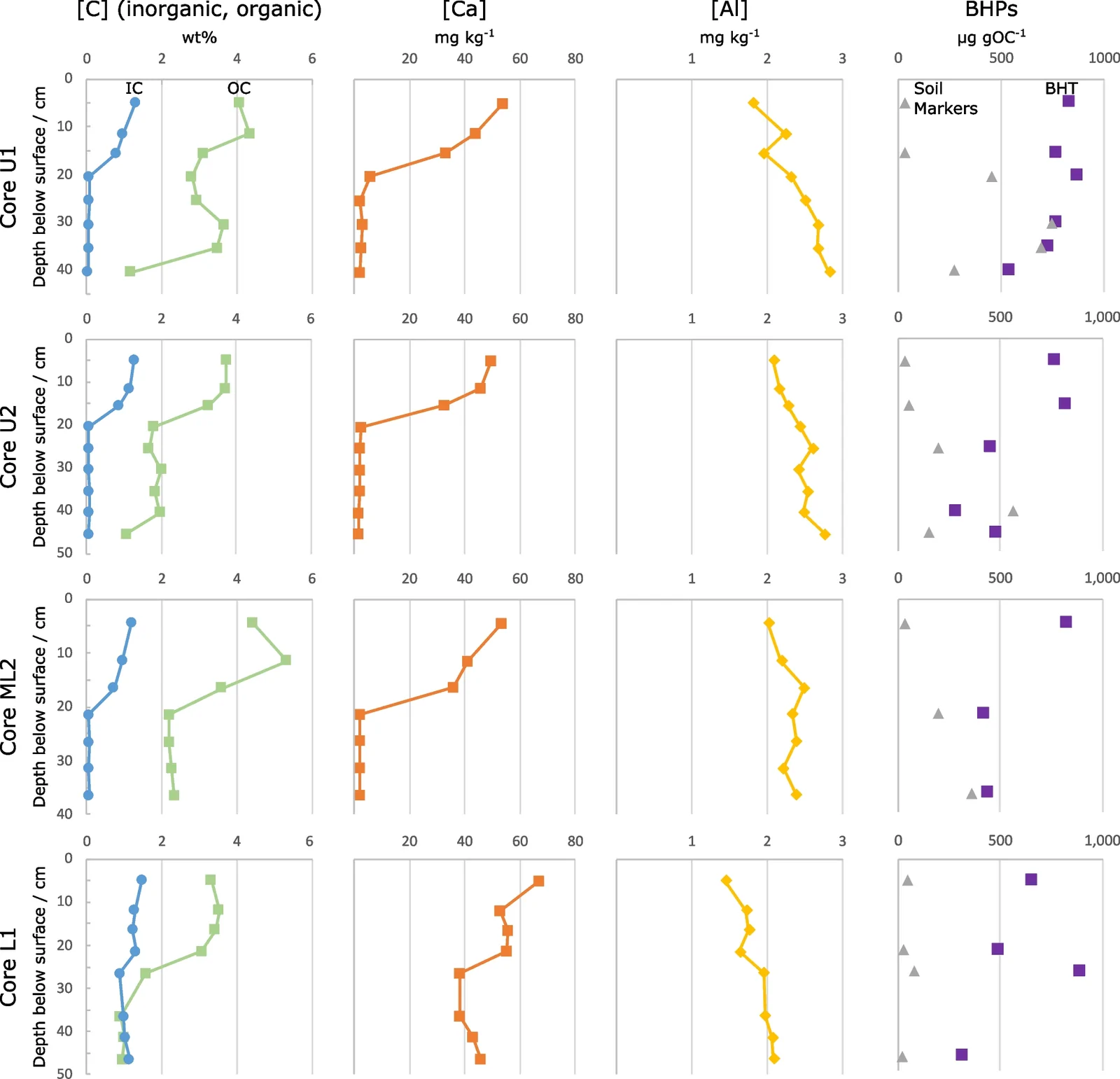
Down-core data showing measured values plotted against depth below sediment surface. In the first column, blue dots show inorganic carbon concentration, green squares show organic carbon. The second and third columns report metal concentrations, orange squares show calcium and yellow diamonds show aluminium. The fourth column reports bacteriohopanepolyol (BHP) concentrations normalised against organic carbon content. Grey triangles show soil marker BHPs, purple squares show BHT, a marine BHP marker. Analytical errors are smaller than the data point markers

Total carbon concentrations ranged from 1.12 to 6.28 wt% (3.31 ± 1.47 wt%). Inorganic carbon concentrations ranged from 0.04 to 1.47 wt% (mean 0.60 ± 0.54 wt%), corresponding to IC fractions of TC ranging from 1 to 55% (mean 17%). OC concentrations ranged from 0.89 to 5.34 wt% (2.71 ± 1.15 wt%). The top 0–15 cm of each core were highest in all forms of carbon – the mean values from the uppermost and lowest sample in each core were 5.19 cf. 1.70 wt%, 1.30 cf. 0.32 wt% and 3.89 cf. 1.38 wt%, (TC, IC and OC respectively; Fig. 2).

### BHP Data from HPLC–MS

#### Total BHPs

The sum of the concentration of the 17 targeted BHPs averaged 1008 ± 385 µg BHPs per gram of organic carbon (µg gOC^−1^; 18.7 ± 11.7 µg BHPs per gram of extracted sediment; µg gSed^−1^) and showed a large variability between cores and downcore (Figs. 2, S1). Total BHP concentration per gram sediment was typically highest at the core top, decreasing with depth (Table S1), although core U1 had maximum BHP concentrations in the 33–38 cm deep subsample. The highest value when normalised to organic carbon, with a maximum of 1869 µg gOC^−1^ (44.6 µg gSed^−1^) was at 33–38 cm depth in core U1. The minimum concentration was 384 µg gOC^−1^ (1.90 µg gSed^−1^) at 44–49 cm in L1.

#### Soil and Marine Marker BHPs

BHT was the most common molecule observed, representing 66 ± 20% of all BHPs (Figure S1). BHT concentration per gram of sediment (1.6 to 23.7 µg gSed^−1^) correlated with OC content (R^2^ = 0.88), with concentrations normalised to OC ranging from 284–890 µg gOC^−1^. Concentrations were highest in the core tops, and lowest at the base of the cores. Nucleoside (soil marker) BHPs, both methylated and non-methylated, were observed in every sample, with adenosylhopane always the most common. Nucleoside concentrations normalised to OC were higher in the lower parts of cores U1, U2 and ML2 (Fig. 2).

### Combining Geochemical Results to Interpret Agricultural and Saltmarsh Layers

Plotting OC and IC against TC showed a bimodal distribution (Figure S2). One group, consisting of the deeper parts of cores U1, U2 and ML2 (n = 15), contained little to no IC and was designated as “agricultural”. In these samples, OC was therefore approximately equal to TC (OC = 0.998 × TC – 0.048, R^2^ = 0.9998). A second group contained 0.72 to 1.47 wt% IC, with no correlation between IC and TC (R^2^ = 0.081) or between IC and OC (R^2^ = 0.018). This group contained the higher parts of cores U1, U2 and ML2, plus all of core L1 (n = 17), and was assigned as “saltmarsh”. Within the saltmarsh group, correlation between OC and TC was strongly positive with a negative intercept due to the presence of IC (OC = 0.956 × TC – 0.898, R^2^ = 0.976). OC and IC values were significantly greater in the saltmarsh group than the agricultural group (P values of 0.023 and < 0.0001 for OC and IC respectively).

Soil marker BHP molecules were rare in the saltmarsh group, always < 10% of the total BHP concentration, but showed markedly increased concentrations in the “agricultural” group, where soil markers represented > 15% of BHPs in each sample (Figure S1). The deepest samples in each core often showed a decrease in soil marker concentrations, similar to or lower than the core top concentration. In core L1, collected closest to a channel, soil marker concentrations remained low throughout. R_soil_ index values ranged from 0.03 to 0.66 (mean 0.22 ± 0.20, n = 18). Low R_soil_ values, denoting a high proportion of marine organic matter, were found in the “saltmarsh” group (mean 0.048 ± 0.014, n = 9).

**Fig. 3:**
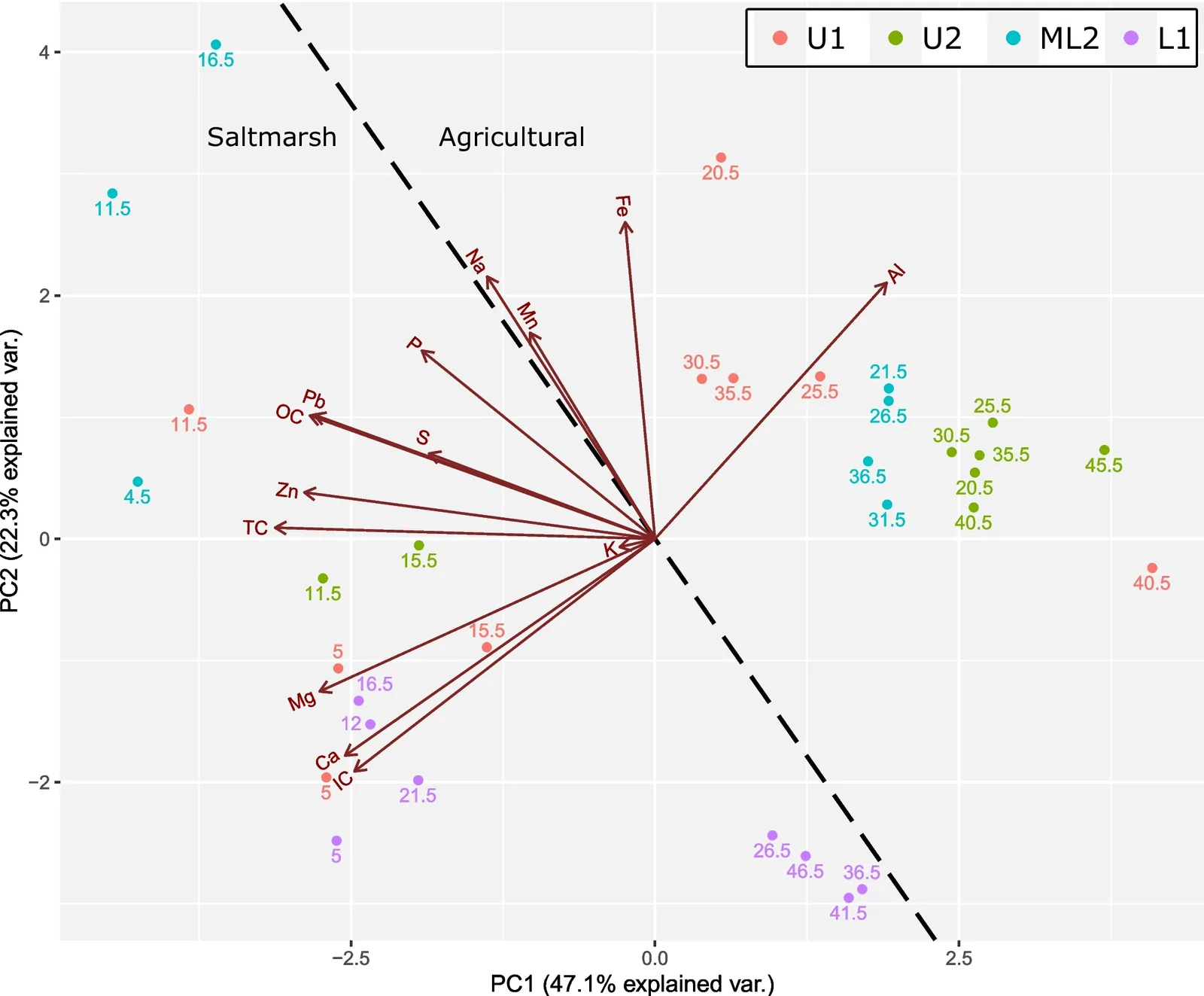
Principal Component Analysis of metal/semi-metal elemental concentrations, total carbon content and the organic and inorganic fractions thereof. The four studied sediment cores are identified by colour. Individual aliquots within each core are labelled by mean depth below sediment surface (cm). Arrows show the vectors of each element/carbon type within the plane of PC1 and PC2. A dashed line, approximately perpendicular to the Ca, Al and IC vectors, splits the dataset into upper and lower parts of the cores. This split is interpreted as separating saltmarsh and agricultural layers

PCA analysis was performed using the following inputs: Al, Ca, Fe, K, Mg, Mn, Na, P, Pb, S, Zn concentrations in mg kg^−1^, TC, OC and IC concentrations in wt%. Principal components 1 and 2 represented 69% of the dataset variance, with good alignment between the IC and Ca vectors, which were opposite to the Al vector (Fig. 3). Mn, Na, P, Pb, S and OC were broadly perpendicular to these three vectors, suggesting that variation in those measurements was not linked to IC, Ca, or Al. A line drawn through the origin, perpendicular to the Ca vector, split the dataset into the saltmarsh and agricultural groups with no errors.

### Defining and Applying Proxy Measurements to Identify Transition Depths

Although elemental concentration data alone (Figs. 2, 3) indicated the transition from agricultural to saltmarsh layers, the absolute concentrations of Ca and IC varied between cores. Within the saltmarsh group, Ca ranged from 33 000 to 67 000 mg kg^−1^ and IC from 0.72 to 1.47 wt%. In the agricultural group, these ranges were 1700—5900 mg kg^−1^ and 0.04–0.07 wt% respectively. Although the twofold variation within the saltmarsh group is relatively small compared to the five- to tenfold increase from agricultural to saltmarsh layers, it blurred the down-core signal when all data was plotted using absolute concentrations. To address this, calcium and carbon concentrations in each sample were normalised against the maximum Ca, IC and OC measurements with the respective core, hereafter referred to as Ca*, IC* and OC* in the text and figures..

**Fig. 4:**
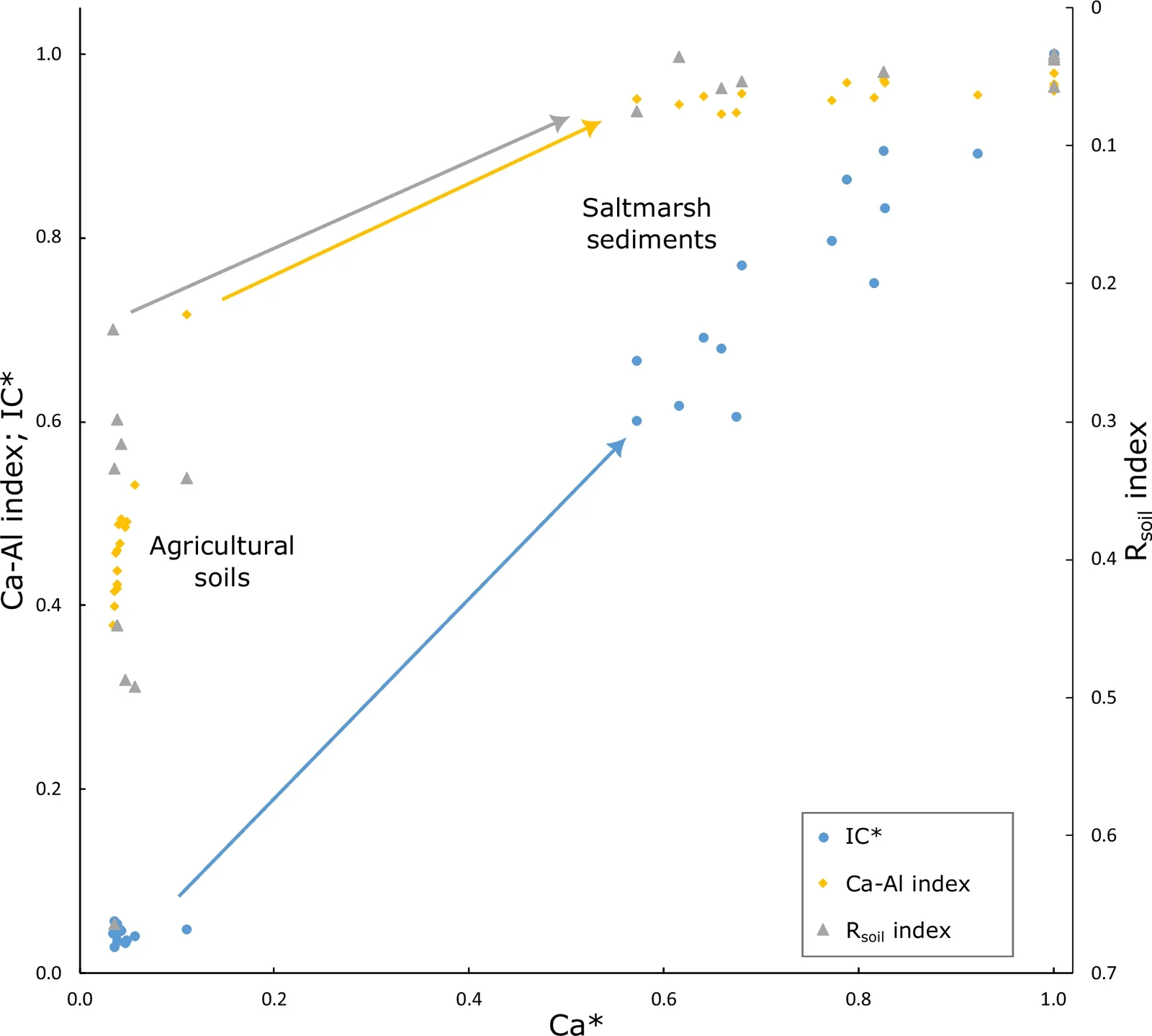
Scatter plot showing the value of different interpreted measurements (blue circles = IC*, yellow diamonds = Ca-Al index, grey triangles = R_soil_ index) compared to Ca* (the relative per-core calcium content). Arrows show the clear distinction between samples lower in the cores, interpreted as pre-restoration agricultural soils and samples higher in the cores representing post-restoration saltmarsh sediments

**Fig. 5:**
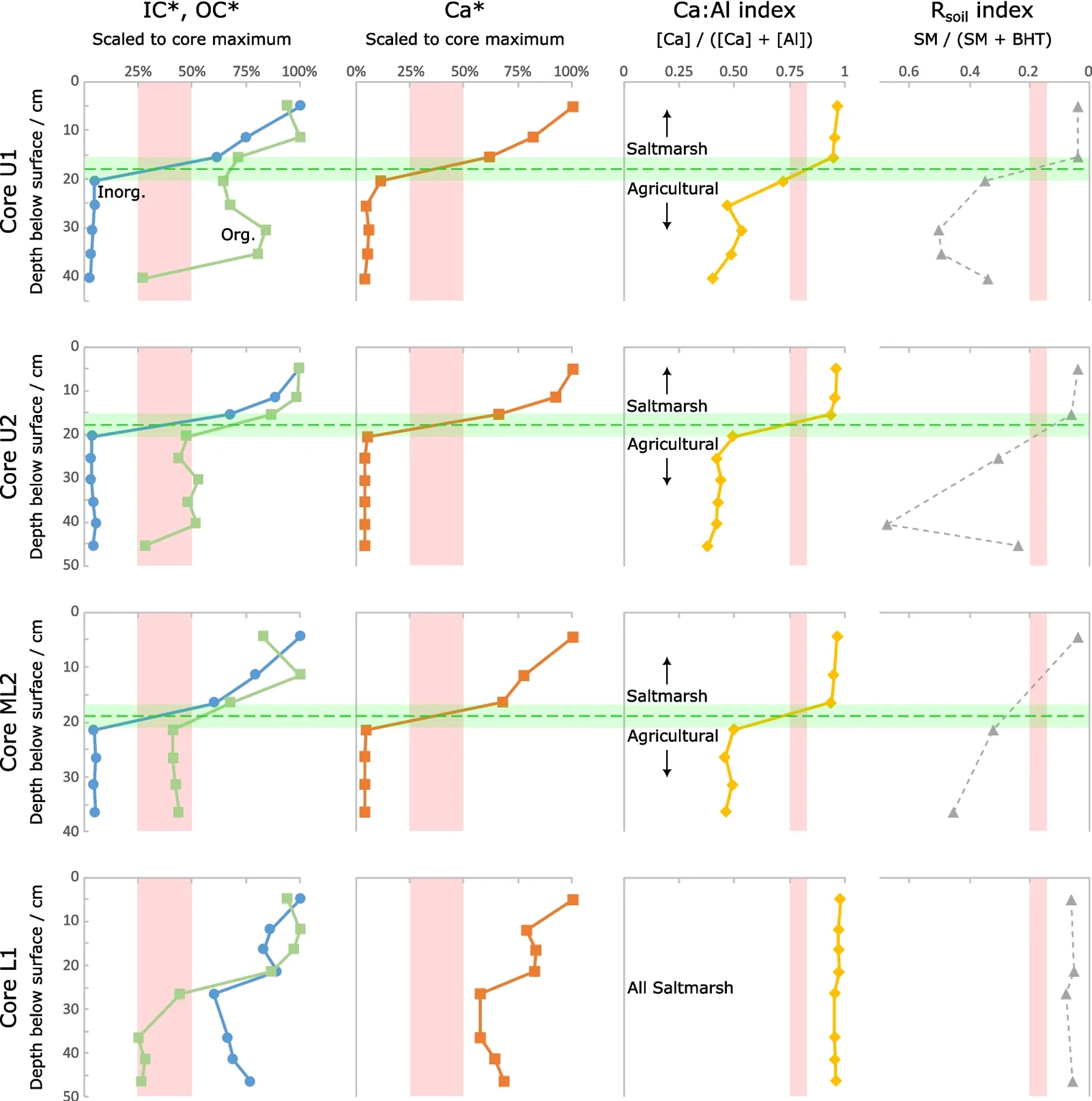
Down-core data showing interpreted values plotted against depth below sediment surface. Blue circles show inorganic carbon normalised against the highest measurement in each core, green squares show normalised organic carbon, orange squares normalised calcium concentrations, yellow diamonds show the Ca:Al index and grey triangles the R_soil_ index. Pink zones show the exclusion zone between saltmarsh and agricultural layers, green horizontal dashed lines show the transition between agricultural and saltmarsh sediment groups. R_soil_ has only dashed lines between data points and does not include the saltmarsh-agricultural boundary interpretation, since not every depth was analysed, but the available data agree with the elemental results

Ca* and IC* values were significantly different (P < 0.0001) in the saltmarsh and agricultural parts of the cores (Fig. 4; saltmarsh Ca* = 0.803 ± 0.146, IC* = 0.786 ± 0.156; agricultural Ca* = 0.043 ± 0.008, IC* = 0.045 ± 0.019). There were no samples with Ca* between 0.11 and 0.57, or with IC* between 0.06 and 0.60. Ca* and IC* showed strong positive correlation (IC* = 1.0141 × Ca* + 0.0022; R^2^ = 0.99). This positive correlation likely reflects the presence of marine-derived calcium carbonate in the saltmarsh sediment. There was weak correlation between Ca* and OC* (R^2^ = 0.40) and between IC* and OC* (R^2^ = 0.35), demonstrating that OC* was not a reliable differentiator of saltmarsh and agricultural layers. Abrupt changes in OC* did not always occur at the same depths as in IC* and Ca* (Fig. 5), this may have been due to high OC in the upper agricultural soil horizon, development of an organic-rich horizon in the saltmarsh sediment following vegetation recolonization, or progressive down-core degradation of OC in the two layers (Piñeiro Juncal et al., 2025).

#### Development of a Calcium:Aluminium Index

Ca* and IC* data required a confident measurement of the maximum downcore value, which may not always be available. To mitigate this, Ca results were combined with Al data because the Ca and Al PCA vectors in Fig. 3 were opposite, generating an index value:

$$Ca:Al index=\frac{[Ca]}{\left[Ca\right]+[Al]}$$

The saltmarsh group showed Ca:Al index values always greater than 0.9 (mean 0.96 ± 0.01), the agricultural group was always below 0.55 (mean 0.47 ± 0.08).

#### Confirmation of Categorisation via the R_soil_ index

BHP data provided an independent test of the agricultural and saltmarsh classifications. In the agricultural soil group, all R_soil_ index values were > 0.2, and in the saltmarsh sediment group the values were all < 0.1. R_soil_ correlated with the Ca:Al index (R^2^ = 0.74). Principal component analysis of interpreted values (Ca*, IC*, OC*, Ca:Al, R_soil_) confirmed that the agricultural soil and saltmarsh sediment groups were clearly different. Ca*, IC*, Ca:Al and (where data was available) R_soil_ vectors aligned along PC1, which explained 82–84% of the variance, whereas OC* pointed 49° to 53° away from the other values (Figure S3A for all samples, S3B for the subset including R_soil_ values).

## Discussion

### Determination of Saltmarsh Sediment Depth and Accumulation Rates at Bleadon Levels

The classification of sediment subsamples into saltmarsh and agricultural groups, tested in Sections. 3.5.2, showed no exceptions downcore. As shown in Fig. 5, no sample from the saltmarsh group was found below an agricultural sample. Thus, estimates of the post-restoration sediment accumulation, and accumulation rate, could be generated. In cores U1, U2 and ML2, the third subsample from the top (13–18 cm or 14-19 cm) was classed as saltmarsh, and the sample below was agricultural. All classification methods agreed with this grouping, though some methods were less confident close to the boundary (e.g. the calcium concentration in the lowest saltmarsh subsamples was lower than at the core top). It should be noted that it was unlikely that field subsampling accidentally sliced the cores exactly at the saltmarsh-agricultural boundary and that each ~ 5 cm subsample was subsequently homogenised in the laboratory. Therefore, the uncertainty in the location of the boundary is at least half of the subsample resolution (i.e. ~ 2.5 cm in each direction). Boundary estimates and uncertainties are shown in Fig. 5. Since the coastline at Bleadon was realigned in 2001, and these samples collected in January 2022, the sedimentation rate estimate from these three cores is 7.6–10.6 mm y^−1^, which is an order of magnitude lower than the rate estimated at the nearby Steart Marshes realignment in the first four years after restoration (Mossman et al., 2022).

#### Higher Sedimentation Rates at Localised Sites

All of core L1 was classified in the saltmarsh group: calcium and IC concentrations were consistent downcore, the Ca:Al index was above 0.9 throughout, and all R_soil_ index values were below 0.1. Thus there has been at least 49 cm of sedimentation at this site since 2001, at a rate exceeding 24 mm y^−1^. Core L1 was described as topographically low and colonised by lower marsh vegetation. It could represent a channel present before the realignment that rapidly filled with incoming sediment – rapid sediment accumulation in areas of low topography has been observed at Steart Marshes (Mossman et al., 2022).

#### Comparison Against Remote-Sensed Data

The sediment accumulation observed in the core L1 geochemistry (> 49 cm) broadly agrees with the remote-sensed estimate generated by averaging all pixels within 10 m of the core (mean 43 cm, range 17–63 cm). However, the sediment accumulation estimates for cores U1, U2 and ML2 (15.5—21.5 cm) are approximately half of those estimated using LiDAR (mean accumulation 37–44 cm, minimum per core 16–31 cm, max 54–60 cm). Reasons for disparity could include interference from standing vegetation, difficulty in aligning LiDAR datasets against fixed reference points, and/or the different point size between sediment cores and LiDAR data (< 10 cm^2^ core area vs. 1 m^2^ pixel area, with accumulation calculated using all points in a 10 m radius surrounding the sample location). The different estimates between remote-sensed and on-site measurements present a challenge when estimating site-wide sediment accumulation, since it is not feasible to collect core samples of sufficient density. Therefore, a combined approach is recommended, whereby site-wide remote sensing is augmented by in-situ measurements, for confirmation and calibration.

### Wider Application of the Geochemical Proxies

To determine whether the chemical proxies for saltmarsh and agricultural soil are applicable beyond Bleadon Levels, we collected pilot data from other sites, although a full investigation was beyond the scope and funding of this project.

#### BHP Ratios

BHP biomarkers were extracted from Steart Marshes, further down the Severn Estuary. Surface samples from both realigned saltmarsh and adjacent natural marsh had R_soil_ values below 0.1, matching the saltmarsh group from Bleadon. Core samples, down to 50 cm in the realigned marsh and 70 cm in the natural marsh, also showed R_soil_ < 0.1. Given the extremely high sedimentation rates at Steart (Mossman et al., 2022), it was entirely feasible that the realignment core did not cross the saltmarsh-agricultural horizon. R_soil_ ratios at recently created Irish saltmarshes (North Bull Island) were also < 0.1 (Akhmetkaliyeva et al., 2025), so the BHP-based classification appears to be applicable across sites. However, the cost and complexity of BHP extractions and measurements limits their use as a routine technique.

#### Calcium and Aluminium

Additional sediment samples were retrieved from the archive at Manchester Metropolitan University: A) a combination of restored saltmarsh, natural saltmarsh and agricultural soils from the Tees Estuary underlain by Sherwood Sandstone (n = 6), B) core samples from natural and realigned marshes in the same estuary (four cores, n = 57, C) agricultural soils from the Humber Estuary underlain by the Flamborough Chalk formation, adjacent to a managed realignment (n = 44), and D) mangrove sediment from West Africa (n = 12).

Saltmarsh samples from the Tees Estuary gave values of 0.92 ± 0.03 (c.f. 0.96 ± 0.01 for Bleadon), so would have been categorised as saltmarsh using the criteria defined above. Agricultural soils from the Tees Estuary gave Ca:Al values of 0.50 ± 0.04 (close to the value at Bleadon, 0.47 ± 0.08), and therefore the Ca:Al ratio would have distinguished them. However, soils from the Humber, overlying chalk bedrock, gave 0.97 ± 0.003, showing that these were dominated by calcium and the Ca:Al index would not be applicable there. Down-core samples from the Tees had high Ca:Al values throughout (0.87 ± 0.06), including in samples that are believed to sit below the pre-restoration horizon, and might be expected to resemble the nearby agricultural samples. This paradox could be explained by the pre-restoration land use. Rather than being agricultural fields, the land here was previously saltmarsh (as evidenced by relic channels in aerial photographs and digital elevation models) and was returned to its previous state by the managed realignments. Thus it is likely that the pre-restoration soils already contained calcium.

As a comparison from a very different coastal ecosystem, 12 sediments from West African mangroves were analysed and showed a contrasting story. Here, the Ca:Al ratio was used to test hypotheses regarding terrestrial vs. marine sediment sourcing. Although these samples were intertidal sediments, there was little calcium present (mean Ca:Al = 0.36 ± 0.08). Accompanying IC data (mean 0.29 ± 0.01 wt%) confirmed that there was little marine carbonate present, and stable carbon isotope results suggested a terrestrial organic carbon source (−26.9 ± 0.8 ‰).

Metal concentrations can be measured easily and relatively cheaply by extraction and ICP-OES, or in-situ using a portable X-ray fluorescence (XRF) analyser. Therefore, the Ca:Al index method could be a valuable screening tool for determining sediment sources – Ca as a general proxy for carbonate minerals, and Al for clay minerals (Zwolsman et al., 1993). Overall, our wider assessment suggested that the Ca:Al index was not universally or consistently applicable, but provided useful information in a range of locations. Future studies could investigate the effect of acid strength on this proxy, since calcium carbonate will dissolve easily compared to aluminium-bearing minerals (Snäll & Liljefors, 2000), and could compare results from ICP-OES and XRF techniques.

## Conclusions and Recommendations

Using new and established proxies, this study showed that reliable geochemical classification of agricultural and saltmarsh sediment layers at Bleadon Levels was possible, without traditional sedimentation records or visible down-core layering. Scaled calcium and inorganic carbon concentrations, normalised against the maximum down-core values, showed clear differences between the agricultural range and saltmarsh range. Quick, simple and cost-effective measurements of calcium and/or inorganic carbon identified the boundary between the two layers (Figs. 4, 5). Normalisation against aluminium using the Ca:Al index confirmed the interpretation and removed the need to calculate absolute concentrations. These measurements could be made in the laboratory, using relatively inexpensive EA, ICP-OES or x-ray fluorescence (XRF) analyses, or even in the field using portable equipment.

Organic carbon concentration measurements were not suitable for separating agricultural soil from saltmarsh sediment. Saltmarsh layers showed reduced OC concentrations down-core in all Bleadon Levels cores (Fig. 2) as in other saltmarsh studies (Akhmetkaliyeva et al., 2025; Beck et al., 2024; Piñeiro Juncal et al., 2025), preventing clear differentiation from the underlying agricultural layer. However, more advanced organic techniques using biomarker molecules showed excellent agreement with the elemental results and further strengthened the interpretations based on elemental techniques.

In the absence of definitive in-situ sedimentation measurements pre- and post-restoration, identification of the thickness of saltmarsh sediment above agricultural soil is challenging. By employing a variety of geochemical techniques at Bleadon Levels we are confident that relatively simple and cost-effective geochemical techniques have accurately identified hidden agricultural soil – saltmarsh sediment transitions.

## Supplementary Information

Below is the link to the electronic supplementary material.

Supplementary file1 Total BHP concentration (black line) and relative contributions (coloured bars) of selected BHPs and groups of BHPs measured in surface sediment samples from Bleadon Levels. The targeted BHPs and groups are (from left to right): anhydro-BHT; unsaturated-BHT; 2-methyl-BHT; 3-methyl-BHT; BHT-cyclitol ether; a group representing all six nucleoside (soil marker) BHPs; BHT. (PDF 40 KB)

Supplementary file2 Scatter plot of organic carbon (OC; orange squares) and inorganic carbon (IC; blue dots) against total carbon (TC) for all four cores. Samples in the “agricultural soil” group are circled in solid brown. Samples in the “saltmarsh sediment” group are circled in dashed blue. (PDF 23 KB)

Supplementary file3 Principal Component Analysis of interpreted data (relative Ca, relative organic C, relative inorganic C, Ca-Al index, R_soil_ index). Data points are split into Saltmarsh (red open circles) and Agricultural (blue dots) groups based on the PCA results shown in Figure 2. Panel A includes every sample but therefore excludes the R_soil_ index, Panel B shows the subset of samples with R_soil_ index values. (PDF 97 KB)

Supplementary file4 (XLSX 26 KB)

## Data Availability

The datasets generated and analysed during this study are included in the supplementary information files.
