# Supplementary material for: How Deep Is Your Mud? Pinpointing Hidden Transitions in Restored Saltmarsh Sediments to Support Robust Assessment of Carbon Stocks
Source: Estuaries Coast. 2026 Sep 24;49(6):200. doi: 10.1007/s12237-026-01812-4 (PMC13612652; doi:10.1007/s12237-026-01812-4)
Supplement: Supplementary file 1 [file 12237_2026_1812_MOESM1_ESM.pdf]

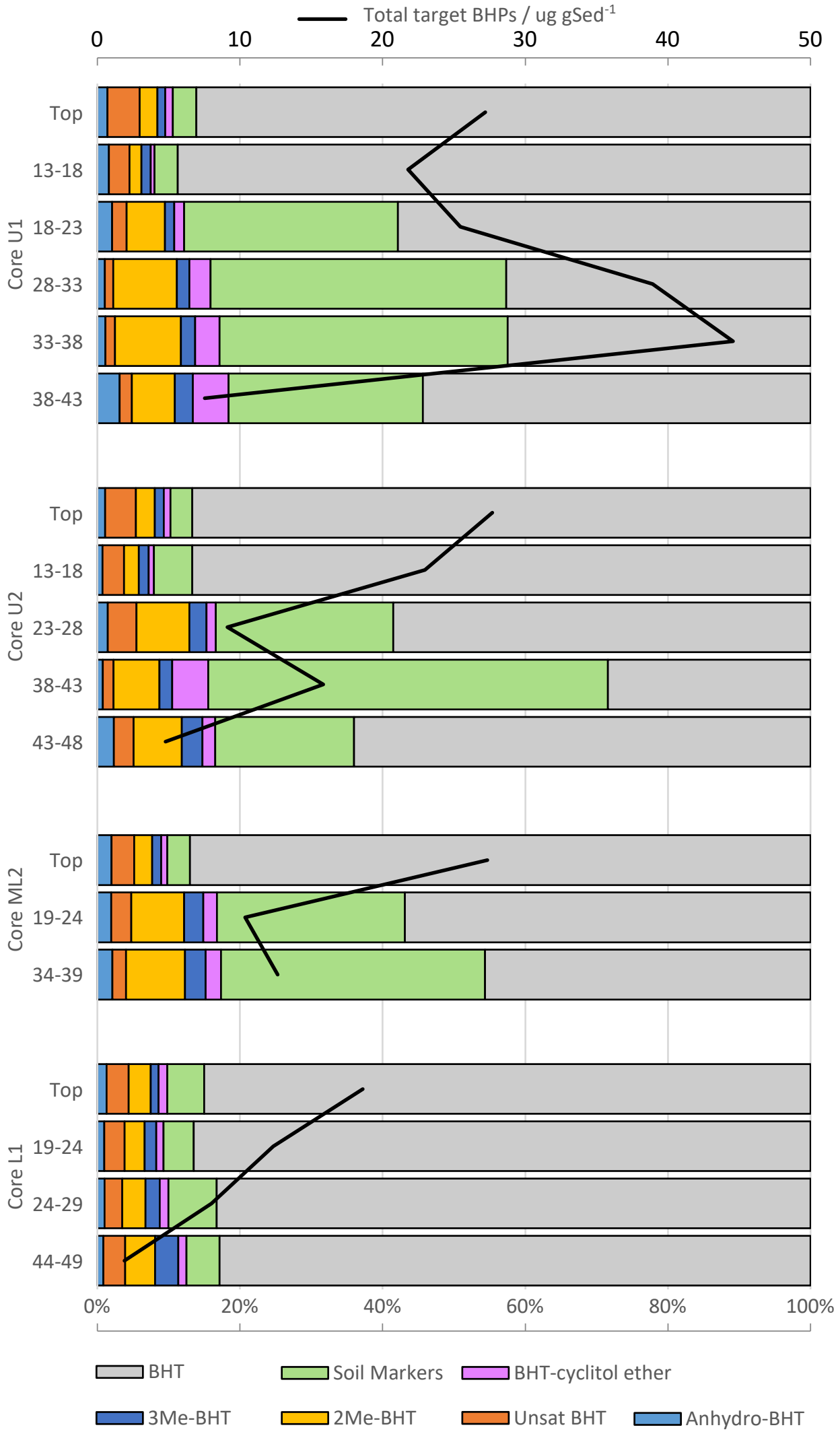

Total target BHPs / ug gSed-1
0
10
20
30
40
50
Core U1
Top
13-18
18-23
28-33
33-38
38-43
Core U2
Top
13-18
23-28
38-43
43-48
Core ML2
Top
19-24
34-39
Core L1
Top
19-24
24-29
44-49
0%
20%
40%
60%
80%
100%
BHT
Soil Markers
BHT-cyclitol ether
3Me-BHT
2Me-BHT
Unsat BHT
Anhydro-BHT
