# Supplementary material for: How Deep Is Your Mud? Pinpointing Hidden Transitions in Restored Saltmarsh Sediments to Support Robust Assessment of Carbon Stocks
Source: Estuaries Coast. 2026 Sep 24;49(6):200. doi: 10.1007/s12237-026-01812-4 (PMC13612652; doi:10.1007/s12237-026-01812-4)
Supplement: Supplementary file 2 [file 12237_2026_1812_MOESM2_ESM.pdf]

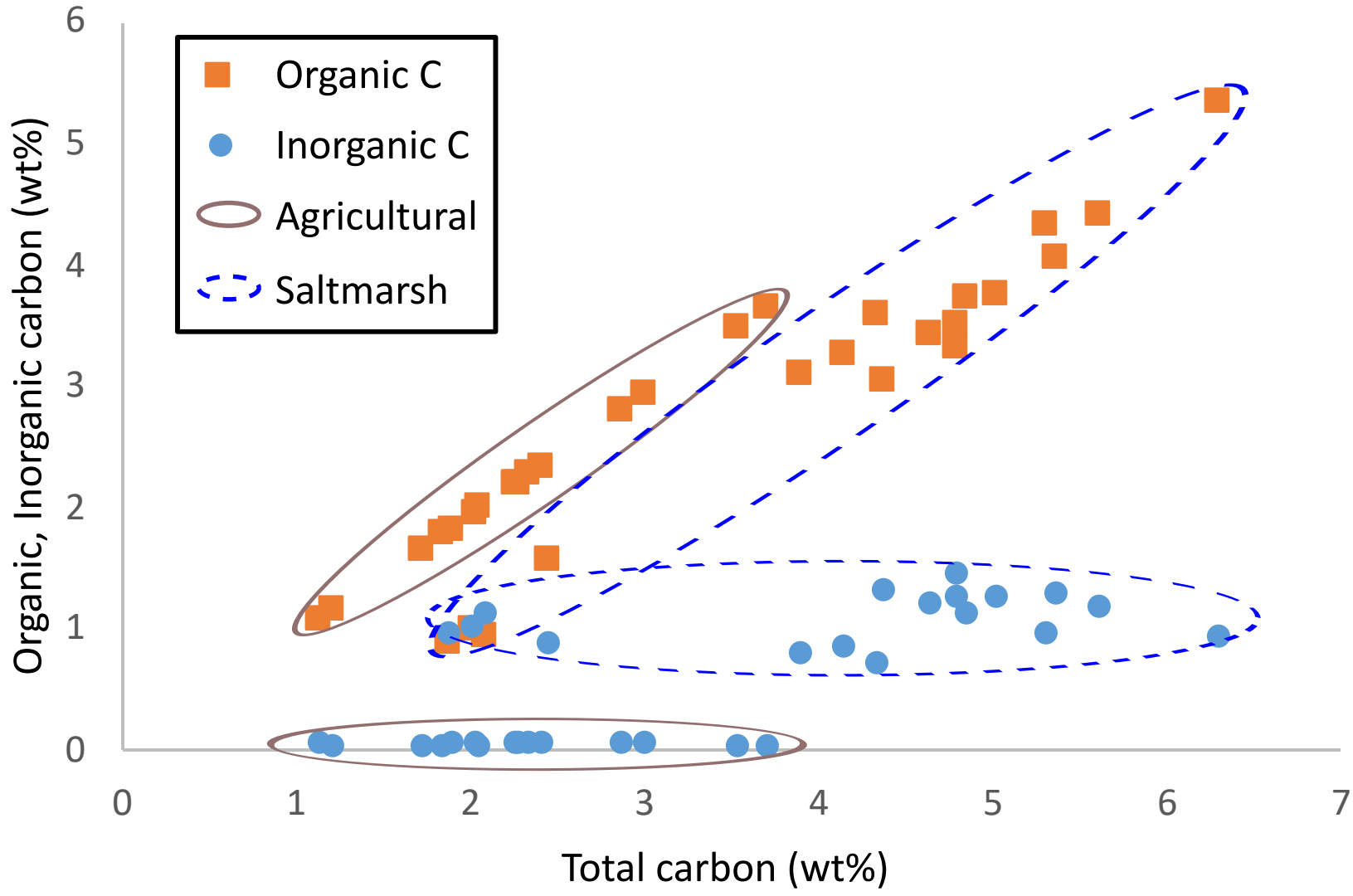

Organic C
Inorganic C
Agricultural
Saltmarsh
Organic, Inorganic carbon (wt%)
Total carbon (wt%)
0
1
2
3
4
5
6
7
