# Supplementary material for: How Deep Is Your Mud? Pinpointing Hidden Transitions in Restored Saltmarsh Sediments to Support Robust Assessment of Carbon Stocks
Source: Estuaries Coast. 2026 Sep 24;49(6):200. doi: 10.1007/s12237-026-01812-4 (PMC13612652; doi:10.1007/s12237-026-01812-4)
Supplement: Supplementary file 3 [file 12237_2026_1812_MOESM3_ESM.pdf]

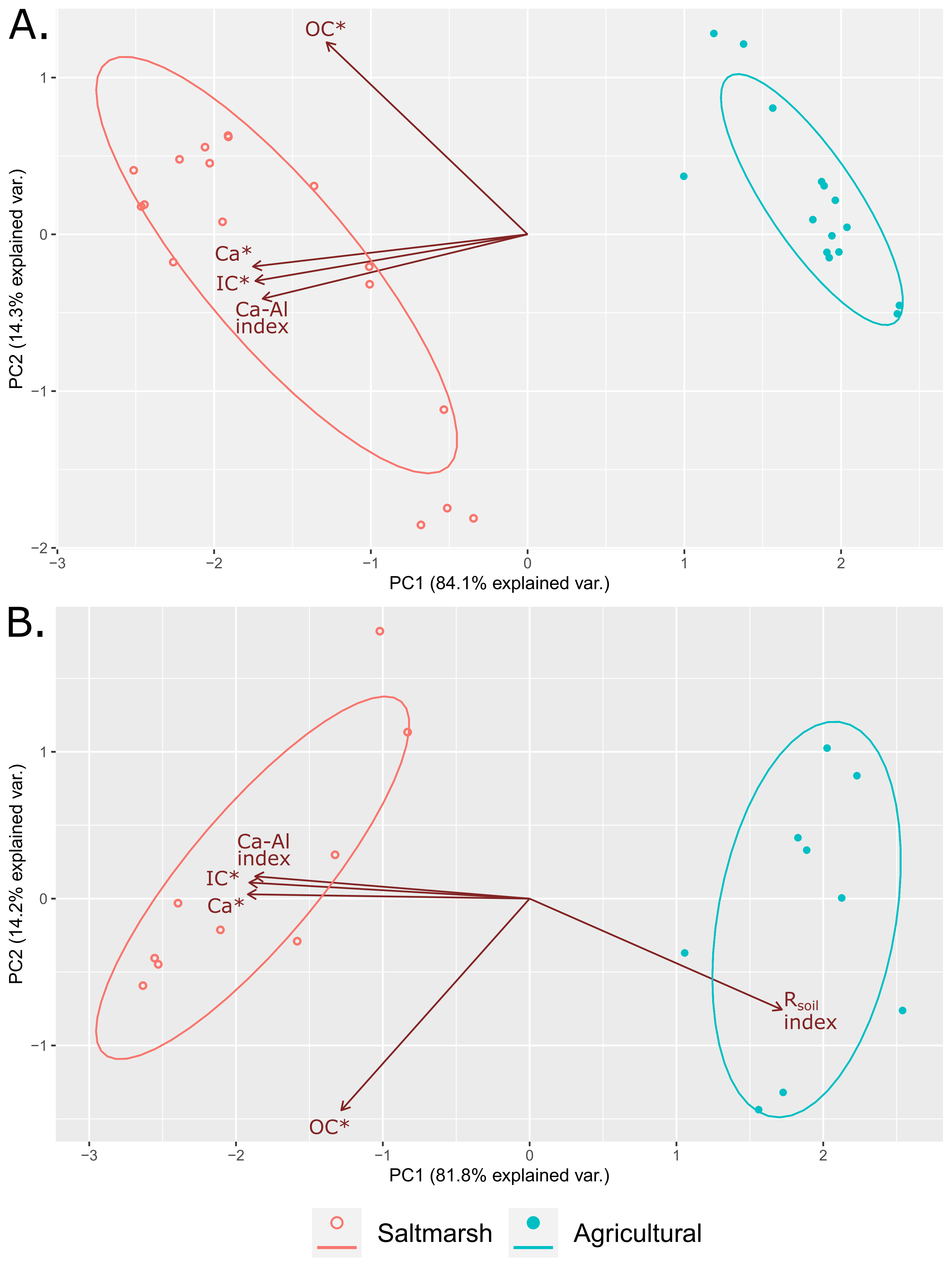
A.
OC*
Ca*
IC*
Ca-Al index
PC2 (14.3% explained var.)
PC1 (84.1% explained var.)
B.
Ca-Al index
IC*
Ca*
R_{soil} index
OC*
PC2 (14.2% explained var.)
PC1 (81.8% explained var.)
Saltmarsh
Agricultural
